# Gene expression of bone morphogenic protein 8B in the primary site, peripheral blood and bone marrow of patients with gastric cancer

**DOI:** 10.3892/ol.2013.1392

**Published:** 2013-06-12

**Authors:** KOSUKE MIMA, TAKEO FUKAGAWA, JUNJI KURASHIGE, YUKI TAKANO, RYUTARO UCHI, HIROKI UEO, TAE MATSUMURA, MASAHISA ISHIBASHI, GENTA SAWADA, YUSUKE TAKAHASHI, SAYURI AKIYOSHI, HIDETOSHI EGUCHI, TOMOYA SUDO, KEISHI SUGIMACHI, MASAYUKI WATANABE, HIDESHI ISHII, MASAKI MORI, HIDEO BABA, MITSURU SASAKO, KOSHI MIMORI

**Affiliations:** 1 Department of Surgery, Kyushu University Beppu Hospital, Beppu, Oita 874-0838, Japan;; 2 Department of Gastroenterological Surgery, Graduate School of Medical Sciences, Kumamoto University, Kumamoto 860-8556;; 3 Gastric Surgery Division, National Cancer Center Hospital, Tokyo 104-0045;; 4 Department of Gastroenterological Surgery, Graduate School of Medicine, Osaka University, Suita, Osaka 565-0871;; 5 Department of Surgery, Hyogo College of Medicine, Hyogo, Honshu 663-8501, Japan

**Keywords:** bone morphogenetic protein 8B, gastric cancer, bone marrow

## Abstract

The prognosis for individuals that are diagnosed with gastric cancer remains poor due to the high frequency of metastatic disease. In response to tumor-derived secreted factors, the bone marrow generates a suitable microenvironment for the development of metastasis. However, it is largely unknown whether secreted factors in bone marrow associated with metastatic disease of patients with gastric cancer are present. Secreted factors from the bone marrow of patients with metastatic gastric cancer were identified using a DNA microarray analysis and the mRNA expression levels were investigated in 355 bone marrow, 295 peripheral blood and 144 primary site samples using quantitative PCR (qPCR). Using DNA microarray analysis, the present study identified bone morphogenetic protein 8B (BMP8B) as a secreted signaling molecule in the bone marrow that was associated with the metastatic disease of human gastric cancer. The expression levels of BMP8B in the bone marrow of 355 gastric cancer patients were increased with metastatic disease. A significant correlation was demonstrated between *BMP8B* mRNA expression in the bone marrow and in the peripheral blood. High *BMP8B* expression in the bone marrow was associated with the diffuse type of gastric cancer (P=0.009), lymph node metastasis (P=0.009), liver metastasis (P=0.044) and peritoneal dissemination (P<0.001). In the primary site, a multivariate analysis revealed *BMP8B* mRNA expression as one of the independent prognostic factors of gastric cancer [hazard ratio (HR), 2.066; 95% CI, 1.132–3.772]. This study suggests that BMP8B, a previously unknown secreted factor in cancer progression, has the potential to be used as a prognostic biomarker. The present study may provide insight into a new mechanism that underlies the dissemination of gastric cancer cells.

## Introduction

Gastric cancer is a worldwide health problem with 876,000 new cases and 647,000 patient mortalities per year (1). Up to one-third of patients with gastric cancer exhibit metastatic disease at the time of diagnosis (2). Although a surgical resection of the tumor remains the primary curative treatment, the recurrence rates are 50–80% (3–5). An improved understanding of the molecular mechanisms of metastatic gastric cancer and the identification of new markers may improve the outcome for affected patients.

Bone marrow is believed to generate a suitable microenvironment for the development of metastasis through a process called metastatic niche formation (6). A high expression of vascular endothelial growth factor receptor 1 (VEGFR-1) and inhibitors of DNA binding-1 (ID1) in the bone marrow has previously been shown to be associated with metastatic gastric cancer, indicating that bone marrow may play an active role in the metastasis of this disease (7,8). Tumor-derived secreted factors, including VEGF-A, lysyl oxidase and transforming growth factor (TGF)-β, affect the bone marrow, leading to the development of metastasis (9–11). However, it is largely unknown whether secreted factors associated with metastatic disease in the bone marrow of patients with gastric cancer are present. Therefore, the present study aimed to identify the secreted factors in the bone marrow that are associated with metastatic gastric cancer.

Bone morphogenetic proteins (BMPs) are members of the TGF-β superfamily. BMPs exhibit a broad spectrum of biological activities in various tissues through a number of processes, including cellular homeostasis and embryonic development (12). Recently, focus has been placed upon two novel findings concerning the *BMP8B* gene. Whittle *et al* identified that BMP8B activates adipocytes and regulates thermogenesis and energy balance (13), while Nieman *et al* disclosed the intimate association between adipocytes and disseminated ovarian cancer cells (14). The present study initially examined whether the expression levels of *BMP8B* mRNA in the primary site, peripheral blood and bone marrow samples that were extracted from a large number of gastric cancer patients were associated with metastatic gastric cancer.

## Materials and methods

### Bone marrow and peripheral blood samples from gastric cancer patients

Bone marrow and peripheral blood samples were collected from 355 gastric cancer patients who underwent surgery between 2001 and 2004 at the National Cancer Center Hospital (Tokyo, Japan). A total of 295 peripheral blood samples, which were paired with bone marrow, were available for the analysis. Approval for the study was obtained from the ethics committee of the National Cancer Center Hospital and documented informed consent was obtained from all patients. The aspirations of the bone marrow and peripheral blood were conducted under general anesthesia prior to surgery, as described previously (7,8). The bone marrow aspirate was obtained from the sternum using a bone marrow aspiration needle and the peripheral blood was obtained through a venous catheter. The first 1.0 ml of bone marrow and peripheral blood were discarded to avoid contamination by the skin. The second collected 1.0 ml of bone marrow and peripheral blood were placed into 4.0 ml ISOGEN-LS (Nippon Gene, Tokyo, Japan) and stored at −80°C until the RNA extraction procedure. The pathological diagnoses and clinicopathological factors were established based on the Japanese classification of gastric carcinoma (15) and the American Joint Committee on Cancer (AJCC)/International Union Against Cancer (UICC) staging system (16).

### Tumor samples from patients with gastric cancer

Primary tumor samples taken from 144 gastric cancer patients were analyzed. All patients underwent a curative resection for gastric cancer between 1989 and 2002 in the Department of Surgery, Oita Prefectural Hospital or in the Department of Surgery, Kyushu University Beppu Hospital (Oita, Japan). The pathological diagnoses and clinicopathological factors were established based on the Japanese classification of gastric carcinoma (15). The resected cancerous tissues were immediately cut and stored in RNA*later* (Ambion, Life Technologies, Carlsbad, CA, USA), frozen in liquid nitrogen and kept at −80°C until RNA extraction. The RNA was extracted using ISOGEN (Nippon Gene) according to the manufacturer’s instructions. The follow-up periods ranged from two months to 12 years, with a mean follow-up time of three years.

### First strand cDNA synthesis and quantitative PCR (qPCR)

The first strand cDNA synthesis and qPCR were performed as previously described (8). The cDNA was synthesized using the SuperScript III Transcriptor First Strand cDNA Synthesis System for RT-PCR (Invitrogen Life Technologies, Carlsbad, CA, USA) according to the manufacturer’s instructions. The process was performed using a LightCycler 480 II instrument (Roche, Mannheim, Germany) and the primers were designed using the Universal Probe Library (Roche) following the manufacturer’s instructions. Glyceraldehyde 3-phosphate dehydrogenase *(GAPDH)* was used as an internal control. The primer sequences and probes that were used were as follows: *BMP8B* forward, 5′-ACCTGGTCATGAGCTTCGTT-3′ and reverse, 5′-AAA GCGGAACTCCTTCCAAT-3′ with universal probe #22; *GAPDH* forward, 5′-TTGGTATCGTGGAAGGACTCTA-3′ and reverse, 5′-TGTCATATTTGGCAGGTT-3′ with universal probe #60. A standard curve was produced by measuring the crossing point of each standard value and plotting it against the logarithmic value of the concentration. The concentrations of the unknown samples were calculated by plotting their crossing points against the standard curve and dividing by the *GAPDH* content.

### Gene expression profiling in the bone marrow of gastric cancer patients by oligo DNA microarray analysis

Oligo DNA micro-array analysis was performed using a 3D-Gene Human Oligo chip 25k (Toray Industries Inc., Tokyo, Japan). The microarrays were scanned using the ScanArray Lite Scanner (PerkinElmer, Waltham, MA, USA) and analyzed using GenePixPro version 5.0 (Molecular Devices Inc., Sunnyvale, CA, USA). The raw data of each spot were normalized by substitution with a mean intensity of the background signal, determined by the signal intensities of all the blank spots with 95% confidence intervals. The raw data intensities that were greater than two standard deviations (SD) above the background signal intensity were considered to be significant. The detected signals for each gene were normalized by a global normalization method (the median of the detected signal intensity was adjusted to 25). This microarray study followed the minimum information about a microarray experiment (MIAME) guidelines issued by the Microarray Gene Expression Data group (17). The microarray data files are available from the NCBI GEO database under the accession number GSE41801.

### Immunohistochemistry (IHC)

The sample processing and IHC procedures were performed as described in a previous study (8). The endogenous peroxidase activity was blocked using 3% hydrogen peroxide and the sections were incubated with diluted antibodies. A subsequent reaction was performed using a biotin-free horseradish peroxidase enzyme-labeled polymer from the Envision Plus detection system (Dako, Carpinteria, CA, USA). A positive reaction was visualized with the addition of diaminobenzidine solution, which was followed by counterstaining with Mayer’s hematoxylin. The BMP8B monoclonal antibody (1:100 dilution; Abnova, Tapei, Taiwan) was used as the primary antibody in the present study.

### Statistical analyses

The *BMP8B* mRNA expression levels were normalized to those of *GAPDH*. The patients were divided into high and low *BMP8B* expression groups for the bone marrow, peripheral blood or primary tumor according to the median expression levels of the *BMP8B* mRNA. The correlation between *BMP8B* expression in the bone marrow, peripheral blood or primary tumor and the clinicopathological factors was analyzed using the χ^2^ test. The expression levels of *BMP8B* in the bone marrow and peripheral blood were not normally distributed (as assessed by Kolmogorov-Smirnov test). Therefore, the Spearman’s correlation coefficient (r) was used to study the association between the expression levels of *BMP8B* in the bone marrow and peripheral blood. The cancer-specific survival rate was calculated using the Kaplan-Meier method and then compared using the log-rank test. The statistical analyses were performed as indicated using a statistical analysis software program (Excel Statistics; Social Survey Research Information Co., Tokyo, Japan). P<0.05 was considered to indicate a statistically significant difference.

## Results

### Gene expression in bone marrow associated with metastatic gastric cancer

To identify the secreted factors in the bone marrow of patients with metastatic gastric cancer, an oligo DNA microarray analysis was performed in order to compare the gene expression in the bone marrow of patients with stage III and stage IV disease. The gene lists were filtered to ensure a four-fold or higher change in the expression levels between the two groups. A total of seven genes were identified to be upregulated in the bone marrow of patients with stage IV gastric cancer compared with those with stage III diseases (Table I). The *BMP8B* gene that encodes a secreted signaling molecule belonging to the TGF-β superfamily was analyzed in the present study, as tumor-derived secreted factors are known to affect the bone marrow, resulting in metastatic progression (6).

### High BMP8B expression in bone marrow is associated with the metastatic progression of human gastric cancer

**Figure 1.**
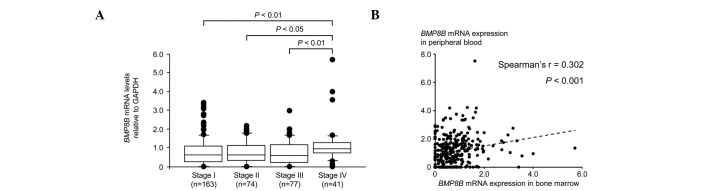
Expression levels of *BMP8B* mRNA in the bone marrow and peripheral blood of gastric cancer patients. (A) The expression levels of *BMP8B* mRNA in the bone marrow of 355 gastric cancer patients by TNM stage. The mRNA expression levels of *BMP8B* were normalized to those of *GAPDH*. The TNM stage was established based on the American Joint Committee on Cancer/International Union Against Cancer (AJCC/IUCC) staging system. Stage I, n=163; stage II, n= 4; stage III, n=77; and stage IV, n=41. P-values were estimated using the Mann-Whitney U test. (B) The correlation between *BMP8B* mRNA expression in 295 paired bone marrow and peripheral blood samples. A correlation coefficient (r) and the corresponding P-value for this correlation were estimated by Spearman’s correlation. BMP8B, bone morphogenic protein 8B; *GAPDH*, glyceraldehyde 3-phosphate dehydrogenase.

To determine the relevance of *BMP8B* expression in the metastatic progression of gastric cancer, *BMP8B* mRNA expression was analyzed in the bone marrow of 355 gastric cancer patients. *BMP8B* mRNA expression was increased in the bone marrow of patients with stage IV diseases (Fig. 1A). With regard to the clinicopathological factors, a significant correlation was demonstrated between high *BMP8B* mRNA expression in the bone marrow and the diffuse type (P=0.009), lymph node metastasis (P=0.009), liver metastasis (P=0.044) and peritoneal dissemination (P<0.001) of the tumors (Table II). *BMP8B* mRNA expression was also examined in the 295 peripheral blood samples that were paired with the bone marrow. A significant correlation was demonstrated between *BMP8B* mRNA expression in the bone marrow and peripheral blood (Fig. 1B; P<0.001; r= 0.30). However, no significant correlation was observed between *BMP8B* mRNA expression in the peripheral blood and the clinicopathological factors (data not shown). These results indicate that high *BMP8B* expression in the bone marrow is associated with metastatic gastric cancer.

### Prognostic significance of BMP8B expression in a primary tumor of human gastric cancer

**Figure 2.**
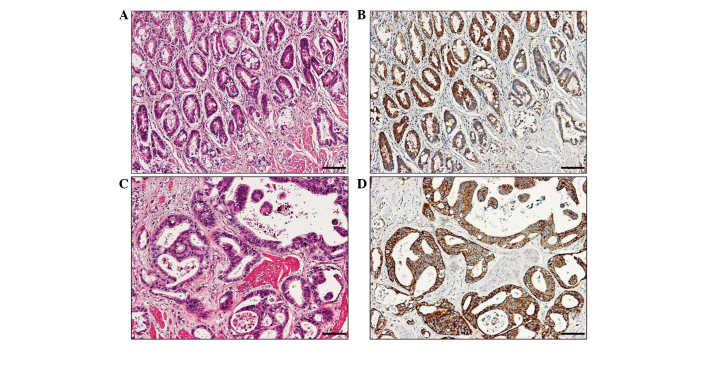
Immunohistochemical staining of BMP8B in human gastric cancer. (A) Hematoxylin-eosin (HE) staining of the gastric mucosa. (B) High BMP8B protein expression in gastric cancer cells infiltrating the gastric mucosa. (C) HE staining of the muscularis propria. (D) High BMP8B protein expression in gastric cancer cells infiltrating the muscularis propria. Scale bars, 100 μm. BMP8B, bone morphogenetic protein 8B.

**Figure 3.**
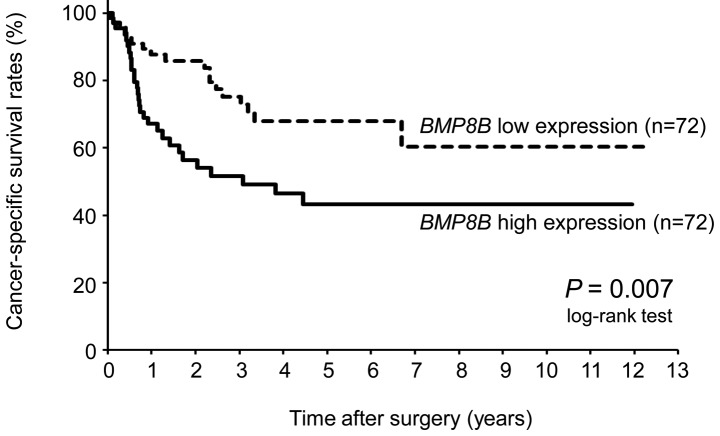
Kaplan-Meier survival analysis of cancer-specific survival in 144 gastric cancer patients comparing the high *BMP8B* expression group and the low *BMP8B* expression group using the log-rank test.

To define the cells that expressed BMP8B proteins in the primary tumors, IHC was conducted on the tumor samples. The BMP8B protein was observed to be expressed by the gastric cancer cells, but not by the stromal cells (Fig. 2). These results indicate that BMP8B is derived from gastric cancer cells in the primary tumor. Next, the expression levels of *BMP8B* mRNA in the tumor samples from the 144 gastric cancer patients were analyzed. The tumor samples from the patients were divided into two groups according to the median *BMP8B* mRNA expression. BMP8B was strongly expressed in the gastric cancer cells of the primary tumors, with high *BMP8B* mRNA expression. This suggested that *BMP8B* gene expression reflects the expression at the protein level. High *BMP8B* mRNA expression in the primary tumor was significantly associated with a shorter cancer-specific survival time following a curative resection (P=0.007; Fig. 3). Furthermore, the multivariate analysis revealed that the prognostic power of *BMP8B* mRNA expression in the tumor was independent of other standard prognostic markers (HR, 2.066; 95% CI, 1.132–3.772; P= 0.018, Table III). There was no significant correlation between *BMP8B* mRNA expression in the primary tumor and the clinicopathological factors (data not shown).

## Discussion

BMPs are members of the TGF-β superfamily. In gastric cancer, BMPs may function as tumor-suppressors or tumor-promoters, depending on the BMP ligands. BMP-2 and BMP-4 suppress the proliferation of diffuse-type gastric cancer cells via the induction of p21 (18). In contrast, BMP-7 expression is associated with a metastatic phenotype and a poor prognosis in human gastric cancer, suggesting that BMP-7 promotes gastric cancer progression (19). To the best of our knowledge, the present study is the first to describe an association between *BMP8B* expression in the bone marrow and metastatic gastric cancer.

The role of BMP8B in cancer is poorly understood. However, it is known that BMP8B activates adipocytes and regulates thermogenesis and energy balance (13). Notably, adipocytes promote the homing of cancer cells to the omentum (14). These results are consistent with the present study, which identified a novel association between *BMP8B* expression and the peritoneal dissemination of gastric cancer cells. Further studies are required to investigate the functional roles of *BMP8B* in metastatic gastric cancer. In the current analyses, the presence of metastatic gastric cancer in the lymph nodes, liver and peritoneum was more closely associated with high expression levels of BMP8B in the bone marrow compared with those in the peripheral blood. BMP8B was expressed by the cancer cells in the primary tumors. Together, these results indicate that tumor-derived BMP8B may affect the bone marrow, leading to metastatic gastric cancer.

In conclusion, the present study revealed that a high *BMP8B* expression level in the bone marrow was associated with metastatic gastric cancer, and that its expression in a primary tumor indicated a poor prognosis. Thus, the results suggest that BMP8B, a previously unknown secreted factor in cancer progression, has the potential to be used as a prognostic biomarker. The present study may clarify a new mechanism that underlies the dissemination of gastric cancer cells.

## Figures and Tables

**Table I.** Comparison between the upregulated genes in the bone marrow of stage III and IV gastric cancer patients.

Symbol	Gene name	Fold change	P-value
AFAP1L2	Actin filament associated protein 1-like 2	5.13	0.003
BMP8B	Bone morphogenetic protein 8b	5.12	0.004
ABCC3	ATP-binding cassette, sub-family C, member 3	5.07	<0.001
ZNF658	Zinc finger protein 658	4.44	0.030
ZNF385D	Zinc finger protein 385D	4.40	0.005
CFHR1	Complement factor H-related 1	4.22	0.005
CD200	CD200 molecule	4.13	0.039

The differences in the gene expression levels between gastric cancer patients with stage III and IV diseases were assessed by measuring the ratios of their expression. A ratio of >4:1 was considered significant. The P-value was estimated using a Student’s t-test to compare the difference in gene expression between these two classes.

**Table II.** *BMP8B* mRNA expression levels of bone marrow and clinicopathological factors in 355 gastric cancer patients.

Variable	*BMP8B* low expression (n=178)	*BMP8B* high expression (n=177)	P-value^a^
Age, years (≤60/>60)	73/105	76/101	0.713
Gender, n (male/female)	114/64	108/69	0.556
Tumor size, cm (≤5/>5)	94/84	85/92	0.367
Histological grade, n (intestinal/diffuse)	169/9	154/23	0.009
Depth of tumor invasion, n (m; sm; mp/ss; se; si)	102/76	94/83	0.427
Lymph node metastasis, n (absent/present)	104/74	79/98	0.009
Peritoneal dissemination^b^, n (absent/present)	170/8	146/31	<0.001
Liver metastasis, n (absent/present)	178/0	173/4	0.044
AJCC/UICC TNM Stage, n (I; II/III; IV)	127/51	110/67	0.066

a Estimated by χ^2^ test. BMP8B, bone morphogenic protein 8B; m, mucosa; sm, submucosa; mp, muscularis propria; ss, subserosa; se, penetration of serosa; si, invasion of adjacent structures; AJCC, American Joint Committee on Cancer; UICC, International Union Against Cancer.

b Peritoneal cytology or positive metastasis.

**Table III.** Multivariate analysis of prognostic factors in gastric cancer following a curative resection.

Variable	HR	95% CI	P-value
Tumor size (>5cm)	1.729	0.920–3.252	0.089
Diffuse type	0.858	0.467–1.574	0.620
Depth of tumor invasion (ss; se; si)	3.575	1.195–10.691	0.023
Lymph node metastasis present	5.321	1.864–15.191	0.002
*BMP8B* mRNA high expression	2.066	1.132–3.772	0.018

BMP8B, bone morphogenetic protein 8B; ss, subserosa; se, penetration of serosa; si, invasion of adjacent structures; HR, hazard ratio.
